# A conceptual framework for effective dissemination and implementation of a policy on school health in rural Nigeria

**DOI:** 10.4102/curationis.v43i1.2100

**Published:** 2020-07-23

**Authors:** Hellen I. Wankasi, Leepile A. Sehularo, Mahlasela A. Rakhudu

**Affiliations:** 1 School of Nursing Science, Faculty of Health Sciences, North-West University, Mmabatho, South Africa

**Keywords:** conceptual framework, dissemination, effective, implementation, policy, school health policy

## Abstract

**Background:**

There is a marked inequality between children in public schools and their counterparts in private schools in terms of school healthcare in rural Nigeria. This is because of the ineffective dissemination and implementation of a policy on school health in public schools. Effective dissemination and implementation of such policy can reduce the prevalence of risky health behaviours amongst young people and have a positive effect on academic performance.

**Objectives:**

The purpose of this study was to develop a conceptual framework for the effective dissemination and implementation of a policy on school health in rural Nigeria.

**Methods:**

This study consisted of four phases as follows: an empirical phase, concept classification, framework development and critical reflection on the conceptual framework. An exploratory, descriptive and contextual research design was used to develop the framework. The work of Dickoff et al. was used to classify concepts from the empirical phase. Chinn and Kramer’s nursing theory on generative method was used for the development of the conceptual framework and for critical review.

**Results:**

The framework consisted of six components, namely, departments of health and education (context); health or educational professionals in the ministries (agents); health or educational practitioners, pupils, parents and communities (recipients); ratification of a policy on school health, stakeholder engagement, training as well as monitoring and evaluation (process); communication, collaborative partnership, commitment and support (dynamics); and effective dissemination and implementation of a policy on school health.

**Conclusion:**

The framework would be a firm foundation and contribution to improve the health of children in public schools, and well-being and academic performance that would be a good feat towards the future endeavour. The findings of the study are pertinent to school health nursing practice, education and research.

## Introduction

A policy means a course of action or direction to follow or take in order to achieve the goals and objectives of an organisation (Joseph 2020:149). School policies, besides managers and school leaders, shape the operations of educational institutions, identify systemic connectivity and integrity, and clarify processes for equitable and inclusive practices (Hourani & Litz 2018:3). Nigeria has a National School Health Policy which was adopted in 2006 with the mission of putting in place adequate resources, facilities and programmes that will guarantee mental, physical and social well-being and the safe and security of the school community that will promote learning outcome of the child (Joseph 2020:149; Sarkin-Kebbi & Kwashabawa 2017:2).

Despite the above information, there is poor dissemination and implementation of a school health policy in Nigeria (Adebayo, Makinde & Omode 2018:9; Dania & Adebayo 2019:504). According to Sarkin-Kebbi and Kwashabawa (2017:8), the school health policy in Nigeria is poorly disseminated and implemented right from Federal, state and local government level. Some of the stakeholders of school health policy are not discharging their duties properly to the extent that some are not even aware of this policy after a decade of formulation. In South Africa, Lenkokile, Hlongwane and Clapper (2016:2) add that not all the schools are disseminating and implementing the school health policy effectively. This is in line with the findings of Rasesemola, Matshoge and Ramukumba (2019:2) who indicated that the implementation of school health policies has been a challenge in sub-Saharan countries such as Nigeria, South Africa, Botswana and Ghana. The absence of policies and/or the lack of proper dissemination and implementation has negative effects on school children (Hourani & Litz 2018:38). According to Adebayo et al. (2018:9), some of the factors affecting effective implementation of the school health policy have been lack of resources, high levels of health misconception amongst teachers and students, low levels of health knowledge amongst trained and practicing teachers, high levels of indifference and negative attitudes amongst non-health teachers. Other factors include ignorance and resistance by school authorities, lack of confidence and incompetence on the part of teachers and head teachers, minimal support from non-governmental agencies as well as lack of legislation to protect school children from health risks in schools (Adebayo et al. 2018:9).

In view of the above concerns, the researchers concur with Dania and Adebayo (2019:505) that there is a need for more research to be performed on the implementation of school health policy. In Nigeria, there is no conceptual framework for effective dissemination and implementation of a school health policy. According to Rossouw, Heyns and Botma (2018:10687), a conceptual framework is made up of sets of concepts that are integrated into significant and meaningful propositions. As such, a conceptual framework is a visual representation of the relationship between concepts derived from existing theories and research. In this study, a conceptual framework development process is similar to a building process, where correlated concepts from the findings of previous studies are linked to create meaning. In view of the above prepositions, there is a compelling need to develop other initiated ideas using a conceptual framework for illustration. Thus, conducting this study becomes imperative to develop a conceptual framework for the effective dissemination and implementation of a school health policy in rural Nigeria.

### Problem statement

The dissemination and inappropriate implementation of a policy on school health is a global concern, including rural Nigeria (Adebayo et al. 2018:9; Dania & Adebayo 2019:504; Sarkin-Kebbi & Kwashabawa 2017:8). In the course of this study, the researcher identified several nursing conceptual frameworks. However, none was related to the dissemination and implementation of a policy on school health. From the researcher’s experience as a school health nurse in rural Nigeria, state ministries conduct orientation activities when staff members are recruited. The content of the programme lacks emphasis with regard to the promotion of school healthcare in the ministries. Thus, stakeholders in the sector demonstrate lack of adequate knowledge of a policy on school health, dissemination and implementation are inappropriate, resulting in education as well as health inequalities amongst children in public schools. To achieve the goal of promoting global school health, there is need to support healthcare professionals and school teachers to equip themselves with knowledge of school health policies through effective dissemination and be more efficient in the implementation of services with new innovation. Based on the above information, it is clear that developing such conceptual framework would facilitate unspontaneous rational decision-making relating to the dissemination and implementation of a policy on school health rather than instinctively carrying out activities that could be detrimental to the programme by policy-makers.

### Purpose of the study

The purpose of this study was to develop a conceptual framework for effective dissemination and implementation of a policy on school health in rural Nigeria.

## Research methodology

A qualitative, exploratory, descriptive and contextual research design was used to develop the conceptual framework. A qualitative approach to inquiry was adopted in this study consisting of four stages: the empirical phase, classification of concepts, development of the framework and reflections on the framework.

### Phase 1: Empirical phase

There were two steps in this phase, namely, a systematic review and individual interviews. Five steps used in a systematic review include framing a clear review question, gathering and classifying evidence, conducting a critical appraisal, summary of the evidence as well as conclusion and recommendations. Interviews were conducted with 24 participants who were selected purposively. Participants were aged between 45 and 59 years, and 16 men and 8 women and their qualifications ranged from BSc to PhD.

### Phase 2: Classification of concepts

The concepts in this context are classified, clearly identified and concisely grouped based on the similarities to link relationships with the six survey list elements (agent, recipient, context, process, dynamics and terminus) (Dickoff, James & Widenbach 1968:545).

### Phase 3: Development of the conceptual framework

The six nursing theory-generative steps were utilised as indicated below.

Six nursing theory-generative steps (Chinn & Kramer 2014:214):

Step 1. Structural development of the conceptual framework

Step 2. State the purpose and relevance of the conceptual framework

Step 3. Assumptions of the conceptual framework

Step 4. Definition of concepts

Step 5. Relationship statement

Step 6. Critical reflection of the framework.

### Phase 4: Reflections on the conceptual framework

The critical reflection of the conceptual framework was done according to Chinn and Kramer (2014:219). The critical components that the researcher used to reflect on the conceptual framework are clarity, simplicity, generality, accessibility and importance (Chinn & Kramer 2014:219).

### Trustworthiness

Measures to ensure trustworthiness in this study were obtained through credibility, transferability, dependability and confirmability (Graneheim, Lindgren & Lundman 2017:29–34). To ensure credibility and transferability, relevant sources or databases were sorted, and relevant context(s) and relevant stakeholders (responsible for the global promotion of school health) were interviewed. Participants were engaged for 30–45 min, during which data were collected to a point of saturation. The development of the conceptual framework was done using four phases. It means that different data collection and analysis methods were used. Confirmability and dependability were obtained through member checking of data analysed to ensure that inputs obtained from participants were represented. Furthermore, the services of an independent reviewer and co-coders were utilised in both the systematic review data and individual interview data. The transcripts were handed to co-coders and promoters for audit trail, respectively. Different manuscripts were sent to different peer review journals for publication accredited with the Department of Higher Education (DHE).

### Ethical consideration

Ethical approval to conduct the study was obtained from North-West University Health Science Ethics Committee (Ethical clearance number: NWU-00633-18-A9).

## Results and discussion

The results are presented and discussed according to the four phases.

### Phase 1: Empirical phase

There were two steps in this phase, namely, systematic review and individual interviews. The results of systematic review yielded seven themes as follows: information sharing, empowerment of key role players, programme development, commitment from key role players, quality improvement, executive support and collaborative partnerships. These results are explained in detail in a manuscript submitted to an accredited journal for publication. There were two stages under individual interviews. The first stage focused on the perceived barriers to the dissemination and implementation of a school health policy in rural Nigeria. This stage yielded two main themes, namely, perceived barriers and perceived positive factors. The focus of the second stage was on dissemination and implementation of school health policy in rural Nigeria: perceived strengths of stakeholders. The findings of this stage yielded two main themes: perceived existing structures and strategies recommended by stakeholders. The results of this phase were used to develop a conceptual framework.

### Phase 2: Classification of concepts

**FIGURE 1:**
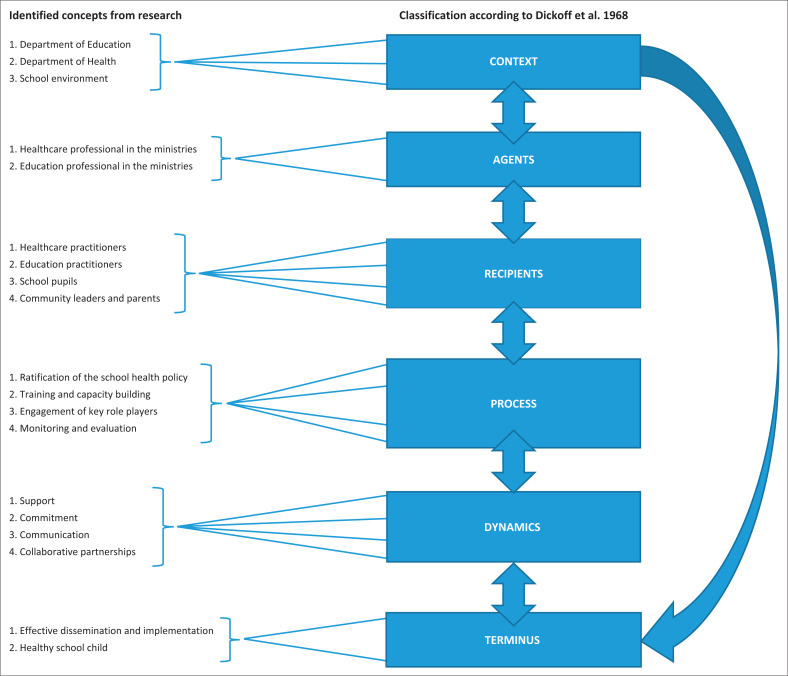
Classification of the concepts according to the survey list by Dickoff et al. (1968).

As indicated in Figure 1, the concepts were derived from the empirical phase and the literature control. The concepts were classified according to the six survey list elements proposed by Dickoff et al. (1968). The elements entailed the agents, recipients, process, dynamics, context and terminus. An agent is a person who can facilitate activities and processes to ensure the effective dissemination and implementation of a policy on school health. Recipients are the main beneficiaries who are to adopt the conceptual framework and those to be impacted by the effective dissemination and implementation of such policy. The context is the environment in which all processes of the framework would take place. The dynamic is the energy source to facilitate the process, which consists of collaborative partnership, communication and commitment as well as support. The process ensures ratification of the school health policy and facilitates other processes such as capacity building, engaging stakeholders, monitoring and evaluation amongst others, to disseminate and implement a policy on school health. Terminus is the effective dissemination and implementation of a policy on school health for school children to be in good health and obtain good academic grades in school. Figure 1 shows how these concepts were classified:

### Phase 3: Development of the conceptual framework

The researcher adapted the six nursing theory-generative steps for the development and description of the conceptual framework (Chinn & Kramer 2014:218). The first item in the theory-generative step consisted of the structural development of the conceptual framework. The researcher demonstrated the structure with a clear diagram of the conceptual framework, which provided general information of the structure at a glance. The description offers general information of the interrelationships of concepts in the conceptual framework, covering the general structure or overview. Other areas considered in this phase are the purpose and assumptions. The next section presents the structural development of the conceptual framework.

### Structural development of the conceptual framework

**FIGURE 2:**
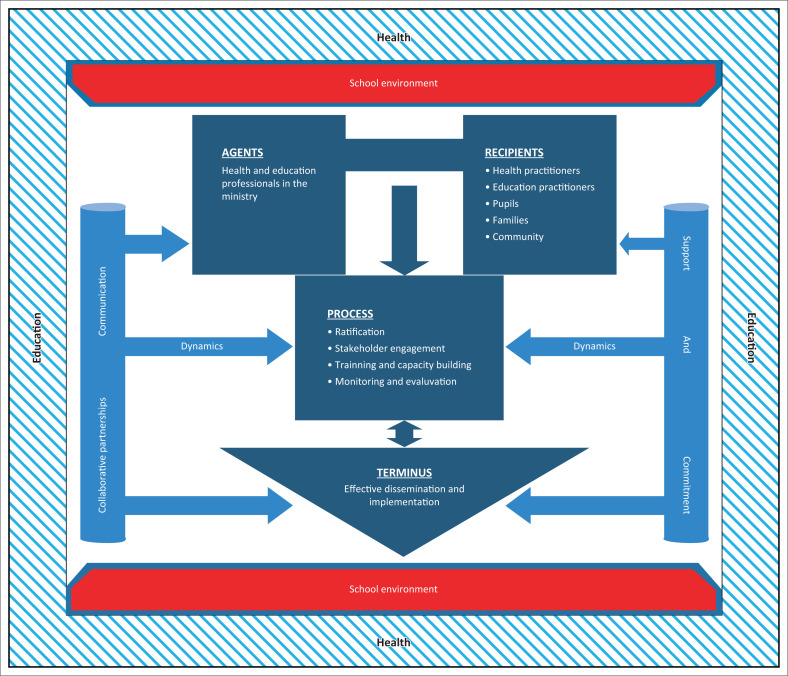
A conceptual framework for effective dissemination and implementation of a policy on school health in rural Nigeria.

Structural development of a conceptual framework is a step-by-step act to support the dissemination and implementation of a school health policy and the facilitation of services. Figure 2 provides a detailed conceptual framework as described by Chinn and Kramer’s (2014) approach. The conceptual framework provides a description of the six survey elements that influenced the process (Dickoff et al. 1968). Figure 2 depicts the conceptual framework of the study.

#### Components of the conceptual framework

The conceptual framework for effective dissemination and implementation of a policy on school health in rural Nigeria has six main elements as follows: educational and healthcare management institutions (context) as institutions facilitating dissemination and implementation; public elementary and secondary school teachers and pupils or students allied to the aforementioned institutions are the users (recipients), teachers and healthcare providers (agents), effective dissemination and implementation of the ratification of a policy on school health (terminus); engagement of role players; capacity building and monitoring and evaluation (process), commitment, communication and support services; and collaborative partnership (dynamics). The different meanings presented are explained as follows:

- The outward edgings around the conceptual framework represent the context in which the dissemination will take place (health and educational institutions), whereas the red structures at top and bottom are the elementary and secondary schools where the implementation would take place.
- The deep blue squared figure at the centre symbolises activities geared towards effective dissemination and implementation of the policy.
- The left side top rectangle represents the agents and the right side rectangle represents recipients of the dissemination and implementation process.

The arrow between the recipient and the agents that points downwards indicates the influence both elements can exert to ensure that the process of dissemination and implementation is conducted.

- The direction of the arrow, from the process to the terminus, symbolises the ultimate goal the framework intends to achieve.
- The protruding (left and right) arrows facing all the elements represents the dynamics (collaborative partnership, commitment, communication and support) needed to influence the agents, recipients and the process for the successful dissemination and implementation of policy outcomes.
- The white coloured background of the conceptual framework indicates a re-awakening of school health promotion activities in rural Nigeria if effectively disseminated and implemented. It also indicates absolute sustainable progressive passion of stakeholders towards accomplishing goals on school health.

#### Purpose and relevance of the conceptual framework

The purpose of this framework is to ensure that the policy on school health is ratified to facilitate activities that would promote effective dissemination and implementation of a policy on school health in rural Nigeria. Thus, the conceptual framework would provide guidance and a reference point to facilitate effective dissemination and implementation of a school health policy.

#### Assumptions of the conceptual framework

Assumptions in this study refer to established realities on which the conceptual framework hinges upon, which add value to the conceptual framework. This conceptual framework was developed from the themes derived from the systematic review and the individual interviews. Amongst others, it was assumed that effective dissemination and implementation of a school health policy requires transforming a policy on school health to a legal document in the state to guide the practice of stakeholders and provide a framework for reference. It is imperative to acknowledge the fact that participation, collaboration and partnership with relevant stakeholders are crucial. These stakeholders include developmental partners, governmental agencies and the role of communities in the dissemination and implementation of a policy on school health for the effective delivery of school healthcare to assist in reducing physical, mental and social problems which school children could develop because of poor implementation. Lastly, that dissemination and implementation are social, collective actions and interactive processes; hence, one variable relies on the other, and no aspect of the conceptual framework is dispensable or stands alone.

**Context:** Context in this study refers to the education, health and school environments under which activities of the framework occur and produce positive results for recipients. One of the participants said: ‘School health policies must be disseminated and implemented by the teachers and school health nurses in health facilities and in our schools’ (P24, Female, 53 years).

**Agent:** Agents in this conceptual framework refer to education and healthcare professionals in the line ministries such as teachers, nurses, public health practitioners and medical practitioners. A participant said: ‘School health policies must be disseminated and implemented by the teachers and school health nurses in health facilities and in our schools’ (P24, Female, 53 years). These agents have shared responsibilities and the authority to ensure ratification of a school health policy to an existing law and to facilitate activities with regard to the dissemination and implementation of a policy on school health. It is therefore expected that these different agents would work together with others to achieve the desired dissemination and implementation goal of a policy on school health in the rural state. A participant said: ‘People should learn to work together; no one can do everything alone’ (P15, Male, 51 years). The different roles of the above agents are described below.

**Teachers:** The first agents needed are teachers. Public elementary and secondary school teachers can play a crucial role in promoting school health in several ways. Teachers detect diseases early when children fall sick, whilst in school, they develop curriculum and impart health knowledge through education. Other motivating factors to explain the importance of teachers are as follows: teachers’ ability to counsel, promote school children’s emotional resilience, conduct school inspection and have the wherewithal to assure quality of school health programmes (Nikolaou & Markogiannakis 2017:11; Sehularo, Manyedi & Du Plessis 2016:227). Consequently, their involvement could create a significant impact on the delivery of school healthcare.

**Nurses:** Nurses have the capability to direct and implement designated school health services within the confines of the state framework. Nurses in this study include school health and public health nurses. Because of the exposure and peculiar training to implement health practice guidelines, nurses advocate, collaborate and work as extension workers to follow up aimed at promoting the health of school children (Sehularo et al. 2016:227).

**Public health practitioners:** Public health practitioners would create a significant impact because they can take up key leadership roles in training, planning and advocate for policy implementation. In respect of human resources, it is important to ensure that the healthcare facilities have sufficient and well-trained health practitioners to ensure effective implementation of a school health policy (Lenkokile et al. 2019:209).

**Medical practitioners:** Involvement of medical practitioners in the effective dissemination and implementation of a policy on school health recognises the mandate of physicians. Moreover, existing structures, available and willing competent personnel in ministries, partnering with other stakeholders to disseminate and implement school health services, would improve the health of school children, their well-being and academic performance.

**Procedure:** The procedure used in this study provides a detailed description on how to conduct activities and safeguard the other five elements (Dickoff et al. 1968:545). The activities are aimed at providing necessary support services from the government on the one hand, and increasing knowledge and awareness of key role players to enable effective dissemination and implementation on the other hand as follows: (1) ratification of a policy on school health into a law to promote the effective dissemination and implementation of a policy on school health; and (2) facilitation of programmes that would promote the dissemination and implementation of such policy. These programmes include training for capacity building, engagement of key role players and monitoring and evaluation, and quotes from participants are also given.

## Ratification of a policy on school health into state law

Formal ratification of a policy on school health into law is inevitable to present the policy as a legal document produced under the authority of state government and undaunted credibility. To ratify such policy into an existing law in rural Nigeria, activities should focus on government actions, create awareness and build knowledge and use existing structures, evidence-based documents and programmes. Government, in collaboration with agents, should facilitate bureaucratic processes to reduce delays and reluctance to endorse and implement a policy on school health. It is expected that ratifying such policy into law would enable the government to provide services, create an enabling environment to motivate and arouse the morale and interest of staff to demand for incentives as well as other motivational activities that would permit the dissemination and implementation of a policy on school health (Hajizadeh 2016:453).

Formal ratification could save the lives of school children through the development of premeditated school health programmes and policies as well as development of personnel proficiencies. Once the policy is ratified, it becomes a legal document, which could serve as a legal framework for advocacy and the protection of stakeholders. Thus, ratification of a policy on school health would serve as a supportive framework for the dissemination and implementation of such policy. In other words, role players are backed up legally if the policy is ratified; thus, they could effectively embark on activities or procedures involving different sectors to ensure the effective dissemination and implementation of such policy.

## Training and capacity building

Capacity building, in this context, entails improvement of skills, proficiencies and capabilities through awareness and knowledge creation with regard to a policy on school health to all relevant stakeholders. One of the participants mentioned that:

‘Even though teachers do teach, I recommend that resource persons should come and train our teachers further to increase their skills and capacities to effectively disseminate and implement school health services/policy by way of workshops and seminars. As you see, some of our teachers, they are not good enough, even the few good ones, if they are not given the opportunity to update their knowledge, the whole previous knowledge would go down the drain.’ (P7, Male, 56 years)

Successful implementation of public policies requires that policy implementers should be appropriately trained in order to attain policy goals or objectives (Lenkokile et al. 2019:208). As it is believed that apt information and communication save life, capacity building programmes could be carried through appropriate information and communication using the print, electronic or sundry media for sensitisation in both non-linear and cascaded approaches to all stakeholders. In addition, capacities could be built through promotional, awareness and knowledge creation activities. Such promotional activities could be viewed as incentives and reimbursements. Studies have revealed that building capacities through incentives and reimbursement of stakeholders would excite interests of key role players in activities that would increase their understanding of the programme towards the dissemination and implementation of a policy on school health (Keothaile 2016). Knowledge creation activities could be conducted in the form of continuous professional development programmes for older staff and orientation or pre-service programmes for the newly employed. Organisers of such programmes could conduct these programmes through seminars, workshops or periodic meetings with the assistance of available human or material resources and agencies.

## Engagement of key role players

Key role players, in this context, refer to as agents holding legitimate interest in the promotion of school healthcare or those with remarkable knowledge in the same subject matter such as teachers, nurses and physicians. One of the participants during individual interviews said:

‘For a school health policy to work, we need to work together, here I’m talking about teachers, nurses and other relevant people, teachers cannot work without nurses and school health nurses cannot work alone without teachers. We must join hands.’ (P10, Male, 58 years)

Collaborations, consultations and involvement of relevant stakeholders could improve effective implementation of a school health policy (Lenkokile et al. 2019:209). Engagement of such agents would promote advocacy, enable them to claim ownership and take charge of the programme as well as follow stakeholders’ resourcefulness. Stakeholders could serve as existing resources to be used by the agent for the benefit of recipients. Engagement of role players for training and capacity building programmes could promote trust, increase understanding and retain positive existing cultures. Others are to promote values, encourage support and reforms or impact as well as help in identifying available resources.

## Monitoring and evaluation

As the process of carrying out activities that would facilitate the dissemination and implementation of a policy on school health evolves, there will be the need for constant monitoring and evaluation at all levels to ensure compliance and that processes are within the frameworks of international best practices with regard to the promotion of school health in terms of processes and outcomes. This statement is confirmed by the following quotation from one of the participants:

‘Then improper monitoring, evaluation and supervision are also affecting all programmes or policies we carry out in our schools. What needs to happen is that we need proper monitoring and evaluation of all policies, including a school health policy we are discussing.’ (P 24, Female, 53 years)

Monitoring occurs when agents are expected to periodically (monthly, quarterly or yearly as the case may be) monitor and evaluate the dissemination and implementation processes with regard to a policy on school health. These would further increase fidelity and effective use of resources and ensure that processes are within prescribed international best standards. Monitoring and evaluation offers the first reliable source of evidence that demonstrates the progress of dissemination and implementation that would ultimately uphold sustainable positive health outcome of school children. This could also empower all agents and recipients to make positive decisions that would affect service delivery in order to promote general health and well-being as well as create a conducive learning environment for teaching and learning.

### Recipient

Recipients are stakeholders in the rural areas of Nigeria, environmentally and strategically situated to interact with related agents to effectively implement a policy on school health and benefit from the process. Recipients rely on the agents, especially desk officers and resource providers to acquire the requisite knowledge, skills and proficiencies as well as motivation for the effective dissemination and implementation of such policy. Healthcare and education practitioners, school pupils, members of the family and the community are also included in the framework. One of the participants mentioned that:

‘[*S*]chool health policies are developed for the school children, their families and the community at large, all of us, must work together to make sure that these policies are a success.’ (P6, Male, 55 years)

### Dynamics

In this regard, collaborative partnership, communication and commitment as well as support from both government and non-governmental agencies (UNICEF), who can render the necessary technical and financial support, could give impetus to the dissemination and implementation of a course on school health. A participant said: ‘We need to utilise available partners for the school health policy to be disseminated and implemented well. For example, then, we can use our school children where applicable’ (P2, Male, 48 years). Another participant said:

‘We all have to play our part, we must all be committed, those who have relevant information or maybe about an updated policies, they must inform us, communication and collaboration will take us far.’ (P19, Male, 48 years)

In South Africa, to ensure a successful dissemination and implementation of the school health policy, the Department of Health (DoH) and Department of Basic Education (DBE) are jointly charged with the responsibility of ensuring a smooth and successful implementation thereof (Lenkokile et al. 2019:97).

### Terminus

The terminus is the ultimate purpose of this conceptual framework. Development and adoption of this conceptual framework would benefit core key role-players (agents), pupils and students (recipients), implementing institutions (context) and society in general. One of the participants mentioned that:

‘[*A*]ll of us must benefit from the framework that we are discussing here and this include the students themselves, their families and teachers and everyone who is working with the students in different schools.’ (P14, Male, 51 years)

For core role players (healthcare professionals and trained teachers), this framework would facilitate the dissemination and implementation of a policy on school health. As their skills would be utilised, they would be motivated through capacity building and update knowledge on a regular basis. Pupils and students in public schools would acquire what is needed to improve their school attendance, concentrate, obtain optimal academic grades to fulfil their life goals and assist their families as well as secure the future of the society in general. Implementing institutions can effectively utilise their staff, having built their capacities, champion school health promotion activities and share knowledge and work in unanimity.

## Relationship statements

The next phase of in this conceptual framework development is the formulation of relationship statements (Chinn & Kramer 2014:227). Relationship statements formulated for this conceptual framework for the dissemination and implementation of such policy are given below.

Dissemination and implementation of a policy on school health is influenced by the context wherein implementation is conducted, namely, educational and healthcare contexts. Effective dissemination and implementation with regard to a policy on school health lies on dynamics such as communication, collaborative partnership and commitment as well as provision of support services.

Dissemination and implementation is the integration of evidence-based interventions into practice settings process; thus, it is a dynamic sequential procedure. This dynamic sequential procedure of capacity building, engagement of key stakeholders and monitoring and evaluation could foster effective dissemination and implementation.

Public elementary and secondary school children (pupils and students), healthcare providers and school teachers are at the centrepiece regarding this issue, given the fact that they are the beneficiaries in elementary and secondary schools. Effective dissemination and implementation is important for all relevant stakeholders and institutions.

Dissemination and implementation is continuous and a dynamic process that would promote an environment where teaching and effective learning in good health takes place for the school child to perform optimally. This enduring process fosters communication and collective competencies for effective dissemination and implementation.

## Phase 4: Critical reflection on the conceptual framework

The critical reflection of this framework was done according to Chinn and Kramer (2014:219) to help to clarify how it relates to theory, practice or research. The critical components that the researcher used to reflect on the conceptual framework are clarity, simplicity, generality, accessibility and importance (Chinn & Kramer 2014:219).

### Clarity

Clarity relates to the structural traits and consistency of the conceptual framework. Clarity of the framework was achieved through the systematic review and empirical data obtained from participants perceptions. The six elements in the survey list were used as the basis for developing the conceptual framework, which made it precise and the content being comprehensive.

### Simplicity

Simplicity refers to the uncomplicatedness of the framework. The structure of the conceptual framework is not too complex. The six survey list of Dickoff et al. (1968:545) made the framework to be simple and easy to follow. Simple English was used in the framework and edited by the professional language editor.

### Generality

The findings of a qualitative study cannot be generalised to other contexts but can be applied. The conceptual framework in this study was developed for schools and health contexts in rural Nigeria. However, the framework can be applied in other contexts outside Nigeria.

### Accessibility

Accessibility is how easy it is to identify elements and the ultimate outcome of the conceptual framework in school health and educational practices. The framework developed in this study will be accessed from the website of the university where the study was conducted. The framework will also be presented at local, national and international conferences and published at a journal accredited by DHE.

### Importance

Importance refers to the extent to which this conceptual framework could add value to school nursing goals of practice, dissemination and implementation of research and education at all levels. This conceptual framework is important as it would benefit core-key-role players–agents, pupils and students–recipients, implementing institutions context and society in general.

## Conclusion

The focus of this study was to develop a conceptual framework for the effective dissemination and implementation of a policy on school health in rural Nigeria. A nursing theory generative method was adopted in the study. Emerging themes drawn from previous studies guided the development of the conceptual framework to facilitate the effective dissemination and implementation of such policy in order to promote optimal health, well-being and attainment of good academic grades by school children. The themes revealed the need for immediate ratification of the policy on school health into existing law in the rural state and the facilitation of programmes to promote the effective dissemination and implementation of such policy. This conceptual framework could contribute to the discourse on a policy on school health policy and global dissemination and implementation of services. The conceptual framework was critically reflected, and revealed that the framework met all evaluation criteria of clarity, simplicity, purpose, accessibility and significance as suggested by Chinn and Kramer (2014:219).

## Limitations of the conceptual framework

Identifying and meeting professional nurses and trained physicians involved in school health in the state was a major challenge and huge limitation. This prevented the researcher from obtaining significant inputs from such professionals.

## Recommendations

There is need for the Nigerian government to reduce bureaucratic obstacles and ensure that policies aimed at improving the well-being of school children are translated into laws expediently. Healthcare workers should ensure that they collaborate to ratify the policy on school health and motive healthcare workers with their dues to facilitate the dissemination and implementation of a policy on school health in rural Nigeria. Stakeholders should collaborate with other relevant government and non-governmental agencies as well as individuals (who are experts) to be used as resource persons for training of newly recruited healthcare workers aimed at promoting school health.

Policy-makers at elementary, secondary, sub-tertiary and tertiary educational institutions should redirect efforts towards continuously conducting training programmes that would improve the competencies of teachers before taking up jobs with particular reference to the promotion of school health through effective dissemination and implementation of such policy. There is need to improve skills and proficiencies of role players through awareness creation and capacity building with the assistance of experts. There is need to further evaluate the conceptual framework across all other geo-political regions of the country and other countries for the effective dissemination and implementation of a policy on school health.

## References

[CIT0001] Adebayo A.M., Makinde G.I. & Omode P.K., 2018, ‘Teachers’ training and involvement in school health Programme in Oyo state, Southwest Nigeria’, *Archives of Basic and Applied Medicine* 6(1), 9–15. 29911693 PMC6002249

[CIT0002] Chinn P.L. & Kramer M.K., 2014 *Knowledge development in nursing: Theory and Process*, Mosby, St. Louis, MO.

[CIT0003] Dania O. & Adebayo A.M., 2019, ‘School health program in Nigeria: A review of its implementation for policy improvement’, *American Journal of Educational Research* 7(7), 499–508.

[CIT0004] Dickoff J., James P. & Wiedenbach E., 1968, ‘Theory in a practice discipline: Part I. practice oriented theory’, *Nursing Research* 17(5), 424–454. 5186886

[CIT0005] Graneheim U.H., Lindgren B.M. & Lundman B., 2017, ‘Methodological challenges in qualitative content analysis: A discussion paper’, *Nurse Education Today* 56(1), 29–34. 10.1016/j.nedt.2017.06.002 28651100

[CIT0006] Hajizadeh M., 2016, ‘Legalizing and regulating marijuana in Canada: Review of potential economic, social, and health impacts’, *International Journal of Health Policy and Management* 5(8), 453 10.15171/ijhpm.2016.63 27694657 PMC4968247

[CIT0007] Hourani R.B. & Litz D., 2018, ‘Juvenile education in Abu Dhabi: Insights from and implications of school policies for educational equity and Inclusion’, *Journal of Correctional Education* 69(2), 33–58.

[CIT0008] Joseph O.O., 2020, ‘Appraisal of the implementation of the national school health policy in secondary schools in Nigeria’, *Academic Journal of Interdisciplinary Studies* 9(2), 149–149. 10.36941/ajis-2020-0032

[CIT0009] Keothaile K.J., 2016, ‘Implementation and outcomes of the school health programme in Ditsobotla’, Unpublished masters dissertation, Witwatersrand University.

[CIT0010] Lenkokile R., Hlongwane P. & Clapper V., 2019, ‘Implementation of the integrated school health policy in public primary schools in Region C, Gauteng Province’, *African Journal of Public Affairs* 11(1), 196–211.

[CIT0011] Nikolaou E. & Markogiannakis G., 2017, ‘The role of teacher in primary school students, mental health promotion’, *Global Journal of Human-Social Science Research* 17(5), 1–16.

[CIT0012] Rasesemola R.M., Matshoge G.P. & Ramukumba T.S., 2019, ‘Compliance to the integrated school health policy: Intersectoral and multisectoral collaboration’, *Curationis* 42(1), 1–8. 10.4102/curationis.v42i1.1912 PMC6407319 30843403

[CIT0013] Rossouw S., Heyns T. & Botma Y., 2018, ‘Towards a conceptual framework to guide the education of paediatric nurse experts’, *Gender and Behaviour* 16(1), 10686–10699.

[CIT0014] Sarkin-Kebbi M. & Kwashabawa B., 2017, ‘Revitalising school health programme for effective school administration in Nigeria’, *International Journal of Tropical Educational Issues* 1(2), 199–211.

[CIT0015] Sehularo L.A., Manyedi M.E. & Du Plessis E., 2016, ‘Substance use prevention programmes among adolescents focusing on resilience as a protective factor: A systematic review’, *African Journal for Physical Activity and Health Sciences (AJPHES)* 22(1–2), 227–241.

